# Nomogram-based risk stratification to analyze the value of receiving postoperative adjuvant therapy after neoadjuvant immunochemotherapy for patients with locally advanced esophageal squamous carcinoma

**DOI:** 10.3389/fimmu.2025.1621607

**Published:** 2025-07-28

**Authors:** Qiuying An, Hongyan Wang, Hui Zhu, Yage Jia, Yibing Liu, Zheng Liu, Jin Yan, Zihan Zhang, Yajing Wang, Ping Zhang, Zhiguo Zhou

**Affiliations:** ^1^ Department of Radiation Oncology, The Fourth Hospital of Hebei Medical University, Shijiazhuang, Hebei, China; ^2^ Department of Thoracic Surgery, The Fourth Hospital of Hebei Medical University, Shijiazhuang, Hebei, China; ^3^ Department of Medical Oncology, The Fourth Hospital of Hebei Medical University, Shijiazhuang, Hebei, China; ^4^ Department of Oncology, Handan Central Hospital, Handan, Hebei, China

**Keywords:** locally advanced esophageal squamous carcinoma, neoadjuvant immunochemotherapy, prognosis, nomogram, risk stratification, postoperative adjuvant therapy

## Abstract

**Purpose:**

To develop a prognosis nomogram for locally advanced esophageal squamous cell carcinoma (LA-ESCC) patients after neoadjuvant immunochemotherapy (NICT) and assess postoperative adjuvant therapy (PAT) value through survival risk stratification.

**Methods:**

We retrospectively analyzed 297 LA-ESCC patients (2019–2023) receiving NICT with or without PAT, randomly divided into the training and validation cohorts. Independent prognostic factors were determined by Least Absolute Shrinkage and Selection Operator (Lasso) regression and multivariate Cox analysis. Progression-free survival (PFS) was compared by the Kaplan-Meier analysis.

**Results:**

The median follow-up time after surgery was 31.67 months (2.23-62.5 months) as of January 25, 2025. The 1-year and 2-year PFS rates were 82.8% and 67.8%. The analysis identified tumor length, tumor thickness reduction rate, surgical method, number of lymph nodes dissected, and ypN-stage as independent prognostic factors. In the training and validation cohorts, the Concordance Index (C-index) of the nomogram was 0.776 and 0.818. The area under the curve (AUC) values for predicting 1-year PFS were 0.823 and 0.899, while the AUC values for predicting 2-year PFS were 0.802 and 0.810, respectively. According to the nomogram, patients were divided into three risk groups (low, medium, and high), and there were significant differences in PFS among the groups (*P*<0.001). Survival analysis showed that PAT significantly improved PFS in the high-risk group (1-year: 53.3% vs. 26.7%; 2-year: 35.6% vs. 6.7%, *P*=0.009), but there was no significant difference in the low and medium risk groups.

**Conclusion:**

The prognosis nomogram can effectively predict the PFS of LA-ESCC patients after NICT. Through survival risk stratification, patients in the high-risk group may benefit from PAT.

## 1 Introduction

Esophageal cancer (EC) represents a globally prevalent malignant neoplasm, ranking as the seventh leading cause of cancer-related mortality worldwide (1). Esophagectomy stands as a pivotal treatment for patients presenting with early-stage or locally advanced esophageal cancer (LAEC). However, surgery alone often results in postoperative recurrence or metastasis for LAEC patients, with rates approaching 50% (2). Recent advancements in neoadjuvant therapy have substantially enhanced prognostic outcomes for LAEC patients (3, 4). Especially following the introduction of immunotherapy, Neoadjuvant Immunochemotherapy (NICT) for LAEC patients has demonstrated distinct therapeutic advantages. According to research, NICT not only achieves comparable pathological complete response (pCR) rates to neoadjuvant chemoradiotherapy (NCRT) (5, 6) but also can mitigate surgical complexities (7, 8). Despite these advancements, the treatment paradigm of esophagectomy following NICT for LAEC still encounters challenges. Recent investigations indicate that the 1-year failure rates remain at 20% for LAEC patients, with local recurrence constituting over 60% of treatment failure patterns (9–11). Consequently, accurately predicting and identifying patients with a high risk of recurrence following surgery, and subsequently administering proactive postoperative interventions, are the urgent problems to be solved.

However, the research on the application of postoperative adjuvant therapy (PAT) after NICT in LAEC patients is limited. Only a few retrospective studies have explored the feasibility of PAT following NICT in LAEC patients (12–15). Some results have shown that PAT may enhance postoperative survival among patients (12, 13, 15) In particular, Feng et al. (13) identified ypT_+_N_+_ patients as a specific subgroup benefiting significantly from PAT, with an increase in the 3-year progression-free survival (PFS) rate by 14.6%. Given the current insufficient evidence, our study aims to identify LAEC patients who may benefit from PAT through prognostic nomogram-based risk stratification, thereby providing an evidence-based guidance for postoperative management.

## 2 Methods and materials

### 2.1 Patient selection

The clinicopathological data were collected for EC patients treated at The Fourth Hospital of Hebei Medical University from October 2019 to November 2023. The following were the inclusion criteria:1) aged between 18 and 80 years; 2) pathological diagnosis of locally advanced esophageal squamous cell carcinoma (LA-ESCC) with clinical staging of cT_3_N_any_M_0_ or cT_1-2_N_+_M_0_; 3) received at least one cycle of NICT before surgery; 4) underwent R0 resection. The exclusion criteria were as follows: 1) history of other malignancies; 2) received anti-tumor treatments other than NICT before surgery; 3) incomplete clinical data or follow-up. The 8th American Joint Committee on Cancer (AJCC) TNM classification system was used in this study. The ethics committee of The Fourth Hospital of Hebei Medical University approved this study.

### 2.2 Variable selection and transformation

The study collected 297 patients who were eligible for enrollment. Comprehensive clinical data were collected for each patient, including age, sex, body mass index (BMI), tumor location, tumor length, the number of NICT cycles undergone, the duration between NICT and surgery, surgical method, ypTNM-stage, and the tumor regression grade (Ryan), et al.

The reduction rate of tumor thickness after NICT is calculated using the formula: [(pre-NICT tumor thickness - post-NICT tumor thickness)/pre-NICT tumor thickness] *100%. Tumor thickness was measured by identifying the thickest cross-section of the tumor in CT images and measuring the distance between the inner and outer tumor margins. When the tumor lumen could not be observed, it was defined as half of the maximum cross-sectional diameter (16). In our study, we used the receiver operating characteristic (ROC) curve to determine the optimal threshold. Additionally, for other discrete variables, if the area under the ROC curve (AUC value) is less than 0.6, we classify using the median value as the threshold.

### 2.3 Treatment methods

All patients received platinum-based doublet chemotherapy combined with PD-1 inhibitor immunotherapy administered every 3 weeks. Common neoadjuvant chemotherapy regimens include platinum (cisplatin or carboplatin) combined with paclitaxel (paclitaxel, nab-paclitaxel, or docetaxel). The PD-1 inhibitors used included tislelizumab, camrelizumab, sintilimab, toripalimab, and pembrolizumab.

The surgical methods include minimally invasive McKeown (MIE-McKeown) esophagectomy, Ivor-Lewis esophagectomy, and Sweet esophagectomy. Lymph node (LN) dissection included three-field lymphadenectomy, as well as complete two-field lymphadenectomy, covering the thoracic, abdominal, and upper mediastinum neck chest junction.

Eligibility criteria for PAT were as follows: 1) patients who did not achieve pCR after NICT; 2) patients with cT_3–4_ and/or cN_+_ stage; 3) ECOG score of ≤2. However, the implementation of PAT is not mandatory. The patients who received PAT in this study started treatment within a timeframe of 4–8 weeks following the surgical procedure. The PAT regimens included chemotherapy combined with immunotherapy (at least 2 cycles), immunotherapy (1–2 years), and radiotherapy-based combination therapy. The prescribed dose of postoperative adjuvant radiotherapy was 50–54 Gy (1.8–2.0 Gy/fraction, 5 fractions/week).

### 2.4 Follow-up and endpoints

The follow-up methods included outpatient records, inpatient registration, and telephone follow-up. The cutoff date of the last follow-up was January 25, 2025. The primary objective of this investigation was to evaluate PFS, which was defined as the interval from the date of the surgical procedure to the date of the first recurrence/metastasis or the date of the last follow-up. According to the classification method of the CROSS study, the failure patterns were grouped into three categories, including Local recurrence (LR), Distant recurrence (DR), and combined local and distant recurrence (LR+DR).

### 2.5 Statistical analysis

A total of 297 patients were randomly divided into the training cohort and the validation cohort (7:3 ratio). The chi-square test was employed to assess the differences in variable distribution between these two cohorts. The prognosis nomogram was constructed based on independent factors determined by the Least Absolute Shrinkage and Selection Operator (Lasso) regression and multivariate Cox regression analysis. Concordance Index (C-index), the area under the curve (AUC) of the receiver operating characteristic (ROC), calibration curve, and decision curve analysis (DCA) were used to evaluate the predictive prowess and clinical utility of the nomogram. To reduce overfitting bias, Bootstrap resampling was performed 1,000 times. X-tile software was used to determine the optimal cut-off value of low-risk, medium-risk, and high-risk stratification in 297 patients. Comparisons of PFS among these groups were facilitated by the Kaplan-Meier method. All statistical analyses of the study were performed by SPSS 26.0 and R-Studio 4.1, and *P*<0.05 in a two-tailed test was considered statistically significant.

## 3 Results

### 3.1 Clinical characteristics and survival of patients

The study included 297 patients, with a median age of 64 years (42–77 years). The median tumor length was 5cm (2-14cm), the median number of NICT cycles was 2 (1–6 cycles), and the median interval between NICT and surgery was 6 weeks (3–16 weeks). Additionally, the median number of LN dissections performed was 24 (2-69). A ROC curve analysis was conducted on the reduction rate of tumor thickness, revealing an AUC value of 0.74 (95% CI: 0.677-0.803), with an optimal cutoff value of 26%. Furthermore, 130 patients (43.8%) received PAT, among whom 101 patients (77.7%) underwent chemotherapy combined with immunotherapy, 16 patients (12.3%) received immunotherapy, and 13 patients (10.0%) accepted radiotherapy-based combination treatment. Additional general characteristics are presented in **Table 1**.

**Table 1:** Comparison of baseline characteristics between the Training cohort and the Validation cohort.

Variables	All patients	Training cohort	Validation cohort	p-Value
	N=297(%)	N=207(%)	N=90(%)	
Age				0.500
<65 years	154 (51.9)	110 (53.1)	44 (48.9)	
≥65years	143 (48.1)	97 (46.9)	46 (51.1)	
Sex				0.811
Male	215 (72.4)	149 (72.0)	66(73.3)	
Female	82 (27.6)	58 (28.0)	24 (26.7)	
BMI				0.919
<18.5	9 (3.0)	6 (2.9)	3 (3.3)	
18.5-24.9	201 (67.7)	139 (67.1)	62 (68.9)	
≥25	87 (29.3)	62 (30.0)	25 (27.8)	
Tumor location				0.637
Upper	23 (7.7)	18 (8.7)	5 (5.6)	
Middle	117 (39.4)	80 (38.6)	37 (41.1)	
Lower	157 (52.9)	109 (52.7)	48 (53.3)	
Tumor length				0.945
<5cm	80 (26.9)	56 (27.1)	24 (26.7)	
≥5cm	217 (73.1)	151 (72.9)	66 (73.3)	
cT stage				0.234
cT1-2	54 (18.2)	34 (16.4)	20 (22.2)	
cT3	243 (81.8)	173 (83.6)	70 (77.8)	
cN stage				0.824
cN0	139 (46.8)	96 (46.4)	43 (47.8)	
cN+	158 (53.2)	111 (53.6)	47 (52.2)	
NICT cycles				0.154
≤2 cycles	167 (56.2)	122 (58.9)	45 (50.0)	
≥3 cycles	130 (43.8)	85 (41.1)	45 (50.0)	
Tumor thickness reduction rate				0.950
≤26%	113 (38.0)	79 (38.2)	34 (37.8)	
>26%	184 (62.0)	128 (61.8)	56 (62.2)	
Time from NICT to surgery				0.975
≤6 weeks	191 (64.3)	133 (64.3)	58 (64.4)	
>6 weeks	106 (35.7)	74 (35.7)	32 (35.6)	
Surgical method				0.520
MIE-McKeown	225 (75.8)	159 (76.8)	66 (73.3)	
Non MIE-McKeown*	72 (24.2)	48 (23.2)	24 (26.7)	
No. of LNs dissected				0.230
≤24	151 (50.8)	110 (53.1)	41 (45.6)	
>24	146 (49.2)	97 (46.9)	49 (54.4)	
Lymphadenectomy				0.931
Three-Field/complete two-field	187 (63.0)	130 (62.8)	57 (63.3)	
No	110 (37.0)	77 (37.2)	33 (36.7)	
Postoperative adjuvant therapy				0.104
Yes	130 (43.8)	97 (46.9)	33 (36.7)	
No	167 (56.2)	110 (53.1)	57 (63.3)	
ypT stage				0.542
ypT0	90 (30.3)	67 (32.4)	23 (25.6)	
ypT1	43 (14.5)	30 (14.5)	13 (14.4)	
ypT2	51 (17.2)	32 (15.4)	19 (21.1)	
ypT3	113 (38.0)	78 (37.7)	35 (38.9)	
ypN stage				0.553
ypN0	175 (58.9)	120 (58.0)	55 (61.1)	
ypN1	79 (26.6)	54 (26.1)	25 (27.8)	
ypN2-3	43 (14.5)	33 (15.9)	10 (11.1)	
ypTNM stage				0.151
0	81 (27.3)	62 (30.0)	19 (21.1)	
I/II	113 (38.0)	72 (34.8)	41 (45.6)	
III/IVA	103 (34.7)	73 (35.2)	30 (33.3)	
Vascular invasion/Nerve invasion				0.502
No	276 (92.9)	191 (92.3)	85 (94.4)	
Yes	21 (7.1)	16 (7.7)	5 (5.6)	
Differentiation				0.670
GX	34 (11.5)	24 (11.6)	10 (11.1)	
G1/G2	113 (38.0)	76 (36.7)	37 (41.1)	
G2-3/G3	60 (20.2)	40 (19.3)	20 (22.2)	
No residual cancer^#^	90 (30.3)	67 (32.4)	23 (25.6)	
Ryan grade				0.552
0	93 (31.3)	69 (33.3)	24 (26.7)	
1	24 (8.1)	18 (8.7)	6 (6.6)	
2	75 (25.2)	51 (24.7)	24 (26.7)	
3	105 (35.4)	69 (33.3)	36 (40.0)	

Non MIE-McKeown*:Ivor-Lewis, Sweet approach.

No residual cancer^#^: ypT0.

The median follow-up time after surgery was 31.67 months (2.23-62.5 months). Among the entire group, 93 patients exhibited recurrence or metastasis after surgery. The 1-year and 2-year PFS rates were 82.8% and 67.8%, respectively. The failure modes of LR, DR, and LR+DR accounted for 67.7% (63/93), 19.4% (18/93), and 12.9% (12/93), respectively.

### 3.2 Prognostic nomogram construction and validation

**Figure 1:**
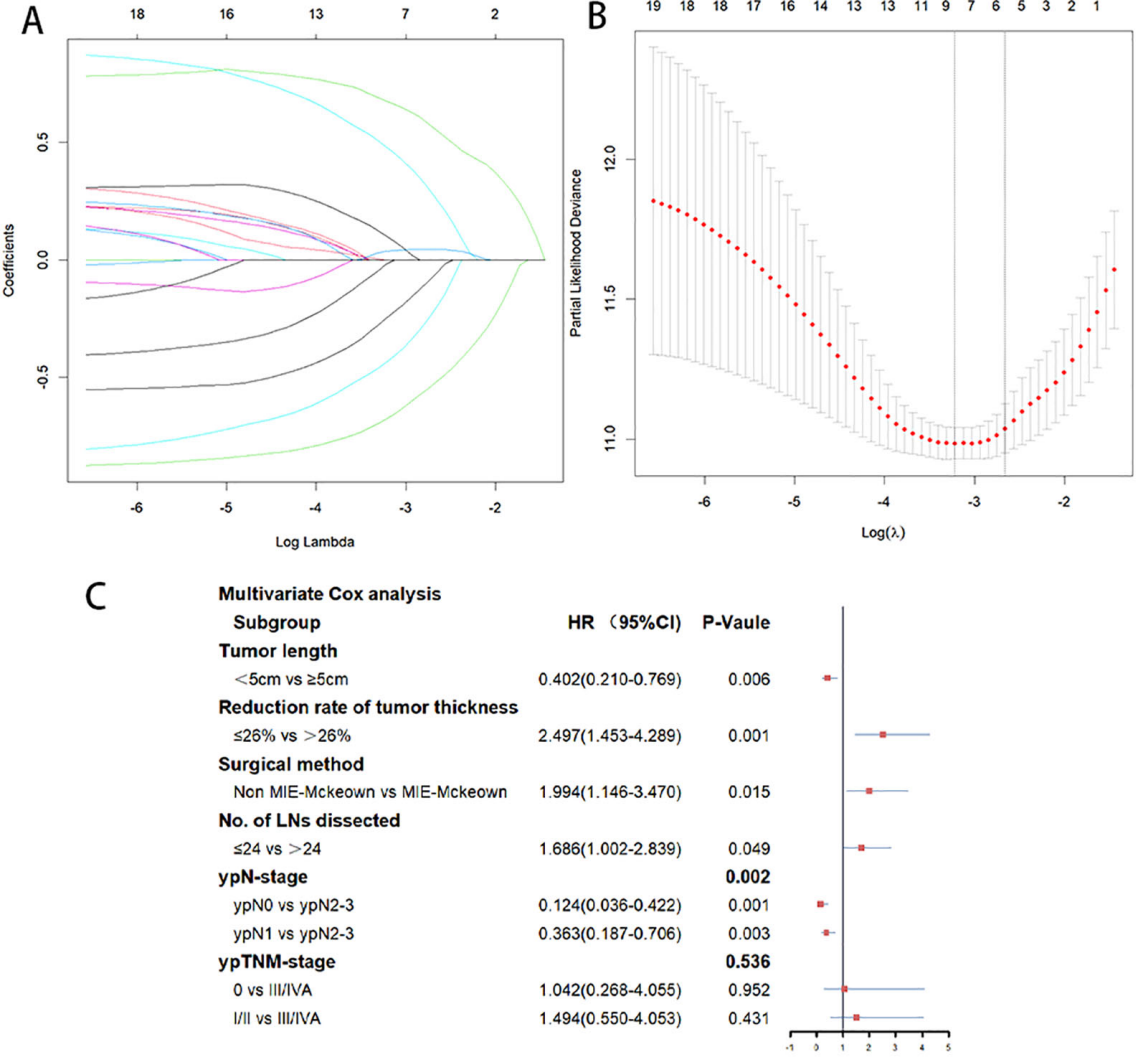
**(A)** Predictors were selected from 20 clinicopathological factors using the Lasso regression method based on the training cohort. **(B)** When the optimal λ was 0.06979749, six factors including Tumor length, Reduction rate of tumor thickness, surgery method, No.of LNs dissected, ypN-stage, and ypTNM-stage were screened. **(C)** Multivariate Cox analysis of the six variables selected by Lasso regression. Tumor length, reduction rate of tumor thickness, surgical method, number of LNs dissected, and ypN-stage were independent prognostic factors influencing PFS.

**Figure 2:**
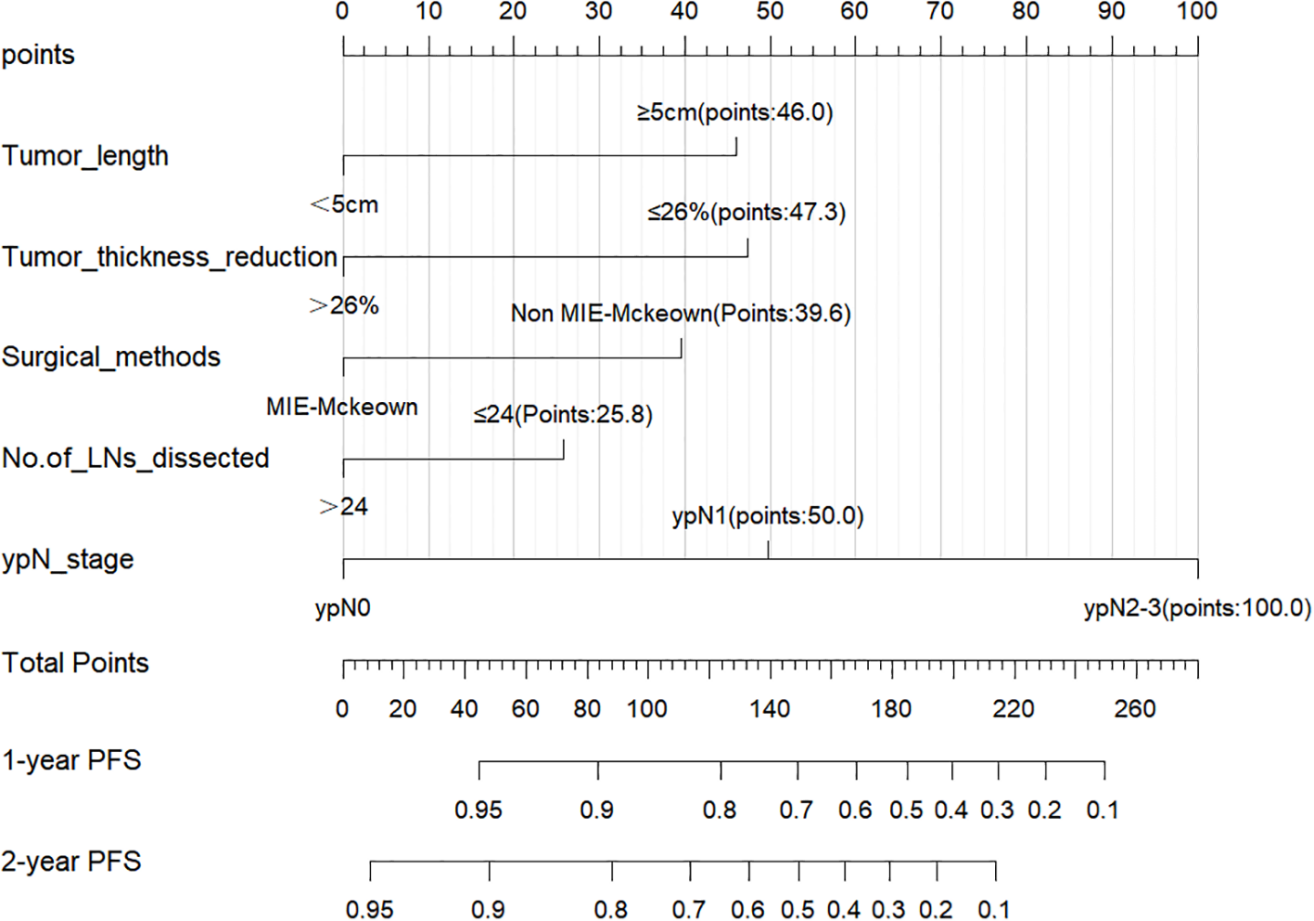
Nomogram for predicting 1-year and 2-year PFS rates of the training cohort. A higher score for each variable in the nomogram indicates a greater risk.

The 297 patients were randomly divided into the training cohort (n=207) and the validation cohort (n=90), and no statistically significant difference was observed in the distribution of variables between the two cohorts (**Table 1**). Based on the training cohort, dimensionality reduction was initially performed using the Lasso regression, selecting variables from the 20 variables (**Table 1**). A total of six variables with non-zero coefficients were identified (**Figures 1A, B**) and subsequently included in a multivariate Cox regression model (**Figure 1C**). The results indicated that tumor length (*P*=0.006), reduction rate of tumor thickness (*P*=0.001), surgical method (*P*=0.015), number of LNs dissected (*P*=0.049), and ypN-stage (*P*=0.002) were independent prognostic factors influencing PFS after surgery (**Figure 1C**). Based on the independent prognostic factors, a nomogram was developed to predict the 1-year and 2-year PFS of patients (**Figure 2**).

**Figure 3:**
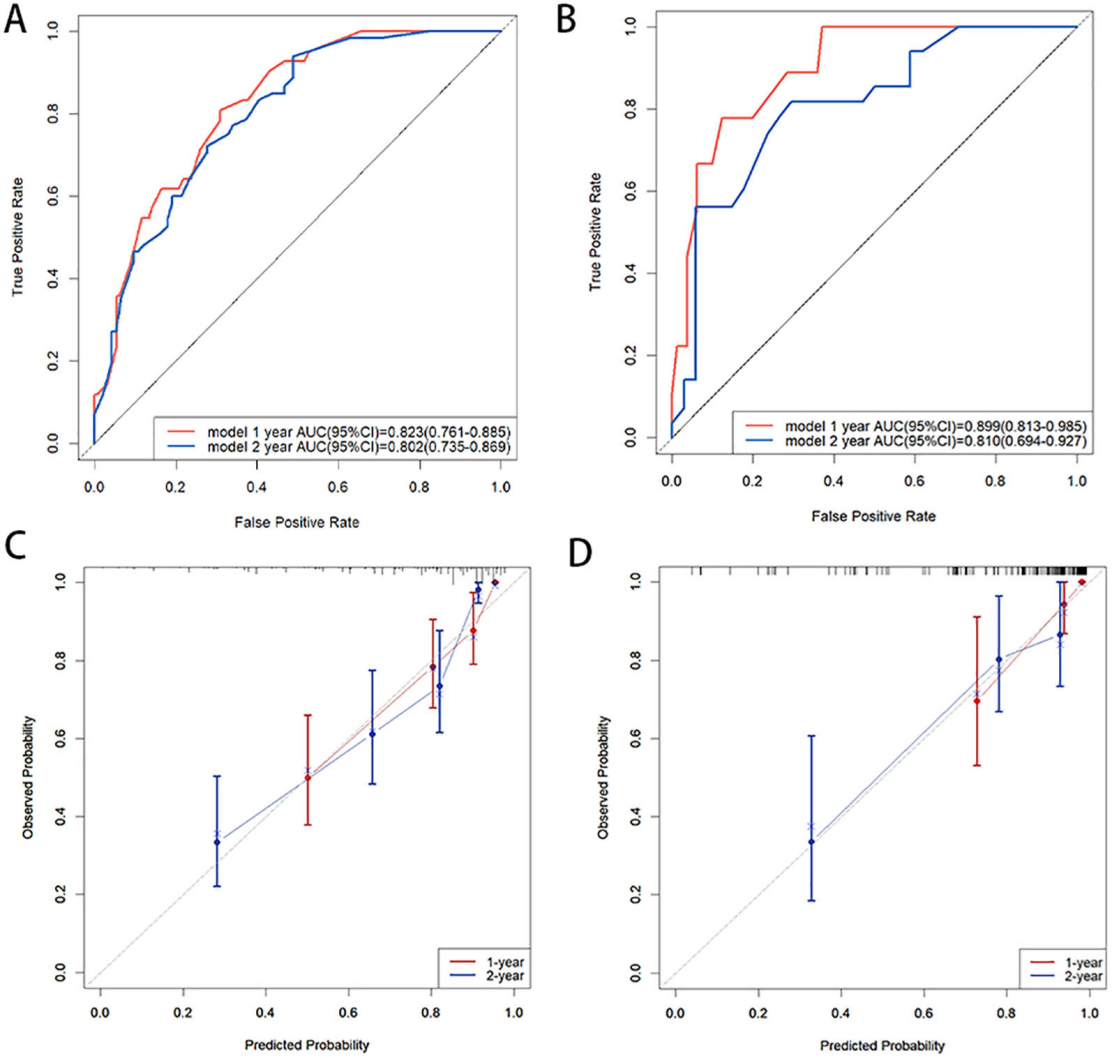
**(A, B)** ROC curves of 1-year and 2-year PFS rates: **(A)** in the training cohort, **(B)** in the validation cohort; **(C, D)** Calibration curves of 1-year and 2-year PFS rates: **(C)** in the training cohort, **(D)** in the validation cohort. ROC curves demonstrated a high discriminative ability of the nomogram, and the calibration curves demonstrated a high concordance between the predicted and actual PFS rates in both the training and validation cohorts.

**Figure 4:**
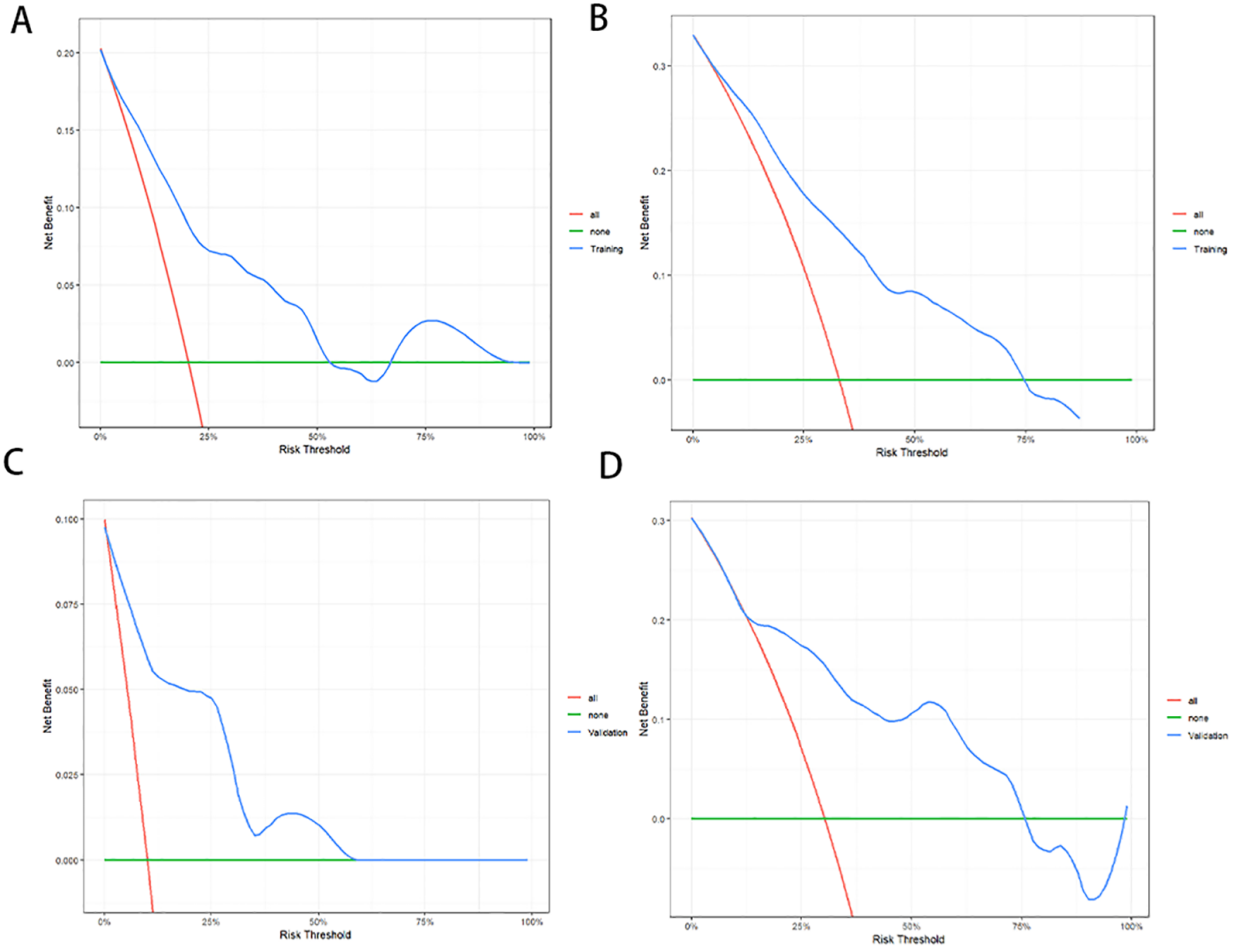
Decision curve analysis (DCA) of the nomogram for PFS prediction. For 1-year **(A)** and 2-year **(B)** PFS in the training cohort. For 1-year **(C)** and 2-year **(D)** PFS in the validation cohort. DCA indicated that the nomogram exhibited good clinical utility in predicting the 1-year and 2-year PFS rates.

In the training cohort, the C-index of the nomogram was 0.776 (95% CI:0.750-0.802). The AUC values for predicting 1-year and 2-year PFS rates were 0.823(0.761-0.885) and 0.802(0.735-0.869), respectively (**Figure 3A**). In the validation cohort, the C-index of the nomogram was 0.818 (95% CI:0.779-0.857). The AUC values for predicting 1-year and 2-year PFS rates were 0.899(0.813-0.985) and 0.810(0.694-0.927), respectively (**Figure 3B**). The calibration curves (**Figures 3C, D**) and DCA curves (**Figures 4A-D**) of the training and validation cohorts further indicated that the nomogram had good predictive performance and clinical utility in predicting the 1-year and 2-year PFS rates after surgery.

### 3.3 Risk stratification based on the nomogram

**Figure 5:**
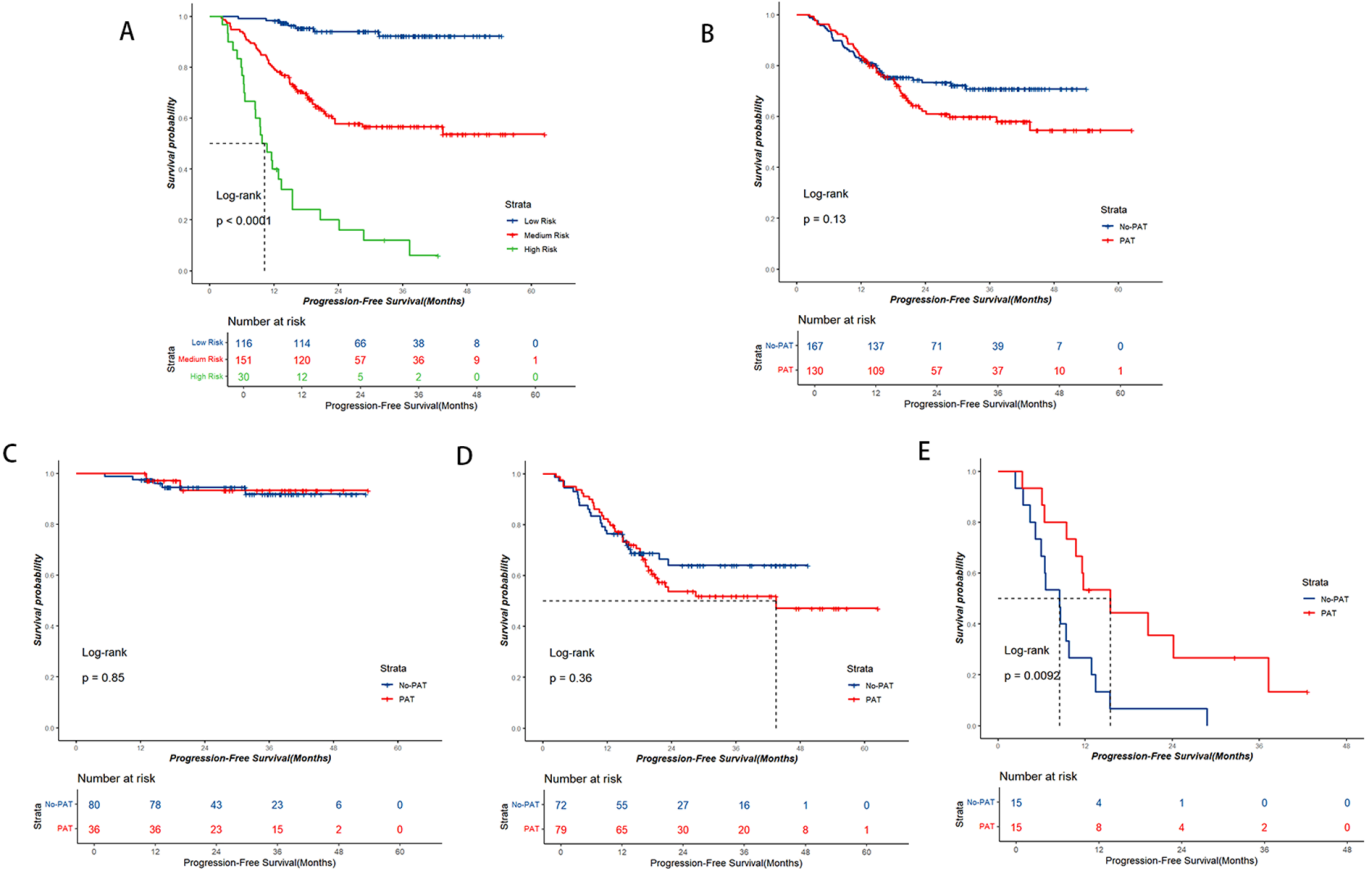
Kaplan-Meier survival curve analysis. **(A)** PFS curve based on nomogram risk stratification. **(B-E)** PFS curves based on the postoperative adjuvant therapy (PAT) status. **(B)** Entire cohort, **(C)** Low-risk group, **(D)** Medium-risk group, **(E)** High-risk group. PAT only exhibited a notable trend of benefit for high-risk patients.

Points based on the independent prognostic factors from the nomogram were individually assigned to each patient, and the total points were calculated. Higher points indicated a greater risk for the patients (**Figure 2**). All patients were categorized into three risk groups: low-risk (116 cases; points ≤ 71.8), medium-risk (151 cases; points:71.8-182.9), and high-risk (30 cases; points>182.9). Survival analysis revealed significant differences in PFS rates among the three groups (1-year PFS rate: 98.3% vs. 79.5% vs. 40.0%; 2-year PFS rate: 94.0% vs. 57.8% vs. 20.0%; **Figure 5A**, *P*<0.001).

### 3.4 The impact of adjuvant therapy on PFS

In the entire group, the No-PAT group had a higher 2-year PFS rate comparable to the PAT group, but no statistically significant difference was observed (2-year PFS rate: 73.3% vs. 62.0%; **Figure 5B**, *P*=0.13). Upon subgroup analysis, within the low-risk patients, the No-PAT group and the PAT group had similar PFS rates (1-year PFS rate: 97.5% vs. 100%; 2-year PFS rate: 94.5% vs. 93.3%; **Figure 5C**, *P*=0.85). In the medium-risk subgroup, no statistically significant difference in PFS rate was observed between the two groups (1-year PFS rate: 76.4% vs. 82.3%; 2-year PFS rate: 64.0% vs. 53.7%, **Figure 5D**, *P*=0.36). However, among the high-risk patients, those who received PAT exhibited significantly better PFS rates, and the 1-year and 2-year PFS rates were improved by 26.6% (53.3% vs. 26.7%) and 28.9% (35.6% vs. 6.7%), respectively, in the PAT group compared with the No-PAT group (**Figure 5E**, *P*=0.009).

## 4 Discussion

In recent years, the application of NICT in LAEC patients has attracted increasing attention. Some clinical studies have confirmed that NICT demonstrates acceptable safety and promising postoperative pathological response rates (7, 8). However, approximately 20% of patients treated with NICT still experience failure within 1 year after surgery (9–11), and the postoperative Local recurrence (LR)rate can be as high as 64.9% (9). Our results were consistent with those reported in previous studies. The 1-year recurrence and/or metastasis rate was 17.2%. In addition, the pattern of failure in our study also observed a high rate of LR, accounting for 67.7% (63/93). The patients who experienced recurrence and/or metastasis after surgery will have a poorer postoperative survival rate (17). Prior research suggested that PAT following NCRT may enhance postoperative survival in patients with a high risk of recurrence, especially among those who did not achieve pCR (non-pCR) (18). However, Xie et al. (14) observed that the non-pCR patients derived no significant benefit from PAT following NICT. We hypothesize that this discrepancy may be attributed to the distinct biological mechanisms of immunotherapy, such as immune modulation and long-lasting immune memory effects (19). Meanwhile, studies have shown that pCR evaluation after NICT is affected by many factors (20, 21). Consequently, relying on postoperative pathology alone to identify patients who may benefit from PAT may not be sufficient. It is essential to establish a multidimensional predictive nomogram for the comprehensive screening of high-risk patients.

Our study revealed that tumor length, tumor thickness reduction rate, surgical method, the number of LNs dissected, and ypN-stage were independent prognostic factors affecting PFS. Notably, tumor length≥5cm has been found to have a notable correlation with the poorer prognosis of patients, which was consistent with the biological characteristics of esophageal squamous cell carcinoma cells (22). We consider that tumor length can directly reflect the longitudinal invasion range of the primary lesion, and indicate an increased risk of LN metastasis when it exceeds a certain length (23, 24). Previous research has demonstrated a close correlation between the dynamic imaging index of tumor thickness reduction rate and the effectiveness of neoadjuvant therapy^-^ (16, 25, 26). In our study, we identified 26% as the optimal cutoff value using the ROC curve, which is marginally higher than the 22% suggested in the NCT cohort by Matsumoto et al (25). We speculate that this difference may be attributed to variations in treatment intensity between NICT and NCT for primary tumors. Furthermore, it was discovered that patients with a high LN metastasis burden (ypN_2-3_) after surgery are significantly associated with poor PFS, which aligns with prior research findings (14, 27). The inadequate tumor response rate and the high LN metastasis burden following NICT elevate the risk of postoperative recurrence and/or metastasis. This may be attributed to the low sensitivity of cancer cells in both the primary tumor and metastatic LNs to neoadjuvant therapy, failing to achieve significant tumor downstaging. Consequently, Wang et al. (28) proposed that preoperative chemoradiotherapy and targeted therapy could be administered to these patients to attain optimal clinical downstaging, ease surgical complexity, and enhance the survival rates of patients.

For surgical quality control in patients with LA-ESCC, our study showed that the choice of open surgery (Ivor-Lewis/Sweet esophagectomy) and the number of LNs dissected less than 24 were unfavorable factors affecting PFS. Previous studies (10, 29) have indicated that LN metastasis in LA-ESCC patients treated with NICT predominantly occurs in the upper mediastinal LNs, specifically in the mediastinal 2R and 4R stations. In clinical practice, compared with open esophagectomy, MIE-McKeown esophagectomy can achieve complete resection of upper mediastinal LNs, particularly those of the right recurrent laryngeal nerve chain (30). In addition, MIE-McKeown esophagectomy can also decrease the risk of postoperative pulmonary infection and other associated complications (31), and minimize the damage of surgical trauma on the patient’s immune system (32). For these reasons, MIE-McKeown esophagectomy is currently a widely used procedure for LAEC patients (33). For the number of LNs dissected, our results were consistent with those reported by Wu et al. (34), which recommended a minimum of 23 LNs to be dissected. Previous studies by Wang et al. (22) demonstrated that LAEC patients are at a high risk of LN metastasis. These metastatic LNs could potentially facilitate distant metastasis by inducing tumor-specific immune tolerance (35), potentially leading to an unfavorable prognosis for patients. Consequently, even after NICT, reducing LN dissection may heighten the risk of postoperative recurrence or metastasis for LA-ESCC patients.

In this study, a prognosis nomogram incorporating the aforementioned five predictors was developed to forecast the PFS of LA-ESCC patients treated with NICT. The C-index of the nomogram was 0.776, while the AUC value exceeded 0.80. Following internal validation, the model demonstrated robust predictive performance and clinical applicability. According to the nomogram, patients were divided into three risk groups (low, medium, and high), and there were significant differences in PFS among the groups (*P*<0.001). Utilizing nomogram risk stratification, we discovered that PAT only exhibited a notable trend of benefit for high-risk patients. Upon further analysis of the risk factors present in the high-risk patient group, it was discovered that all patients in this group exhibited the trait of positive lymph nodes (ypN_+_). Additionally, 93% of these patients showed a tumor thickness reduction rate of ≤ 26%. This finding aligns with the benefit group characterized by ypT_+_N_+_ proposed by Feng et al. (13). Therefore, we consider that ypN_+_ patients exhibiting a poor response to neoadjuvant therapy may constitute the subgroup that derives the most significant clinical benefit from PAT. Previous studies have demonstrated that the presence of tumor cells in lymph nodes not only indicates the metastatic potential of the primary tumor but also enables these cells to colonize and disseminate to distant organs (36). Tumor cells can modify the microenvironment of lymph nodes, leading to an increased accumulation of immune-tolerant or immunosuppressive cell populations within metastatic lymph nodes, which in turn facilitates immune evasion (37–39). Therefore, patients who demonstrate a high nodal metastatic burden following NICT are considered to harbor tumor cells with enhanced metastatic potential and diminished sensitivity to NICT. Despite undergoing surgery, minimal residual disease may persist. Therefore, we speculate that in the high-risk populations, intensified PAT may represent a critical strategy for controlling tumor progression and extending PFS.

At present, the evidence on the selection of PAT regimen after NICT for LA-ESCC patients is extremely limited, and a few studies (13–15) mention that PAT regimens involve immunotherapy combined with chemotherapy, chemotherapy, immunotherapy, and radiotherapy-based combination therapy. Interestingly, some studies found that the subgroup of patients who received postoperative adjuvant chemotherapy alone experienced the poorest outcome. The authors considered that chemotherapy may further impede an already compromised immune system’s capacity to effectively recognize and target cancer cells, ultimately resulting in tumor recurrence (14). Furthermore, given the primary failure mode of local recurrence following NICT, the potential significance of postoperative adjuvant radiotherapy deserves attention. Previous studies indicate that radiotherapy demonstrates a high rate of local control in both neoadjuvant therapy (29) and postoperative adjuvant therapy (40) for esophageal cancer. Consequently, incorporating postoperative adjuvant radiotherapy could potentially enhance the local control rate for LA-ESCC patients who have a high recurrence risk after surgery. Nevertheless, due to the limited number of cases involving postoperative adjuvant radiotherapy in this study (13 cases), further evidence is required.

Our study still has some limitations: 1) This was a retrospective study, which inherently introduces some degree of bias; 2)The fact that it was conducted at a single center without external validation, future research should involve multi-center collaboration to expand the sample size to validate the results; 3) A limited data set for postoperative adjuvant immunotherapy and radiotherapy, lacking further stratification of the treatment protocol; 4) A relatively small number of high-risk cases, making it essential to collect more data or conduct prospective studies for future verification.

## 5 Conclusions

The prognosis nomogram can effectively predict the PFS of LA-ESCC patients after NICT. Through survival risk stratification, patients in the high-risk group may benefit from PAT.

## Data Availability

The raw data supporting the conclusions of this article will be made available by the authors, without undue reservation.
