# Supplementary figures and images for: Nomogram-based risk stratification to analyze the value of receiving postoperative adjuvant therapy after neoadjuvant immunochemotherapy for patients with locally advanced esophageal squamous carcinoma
Source: Front Immunol. 2025 Jul 28;16:1621607. doi: 10.3389/fimmu.2025.1621607 (PMC12336237; doi:10.3389/fimmu.2025.1621607)

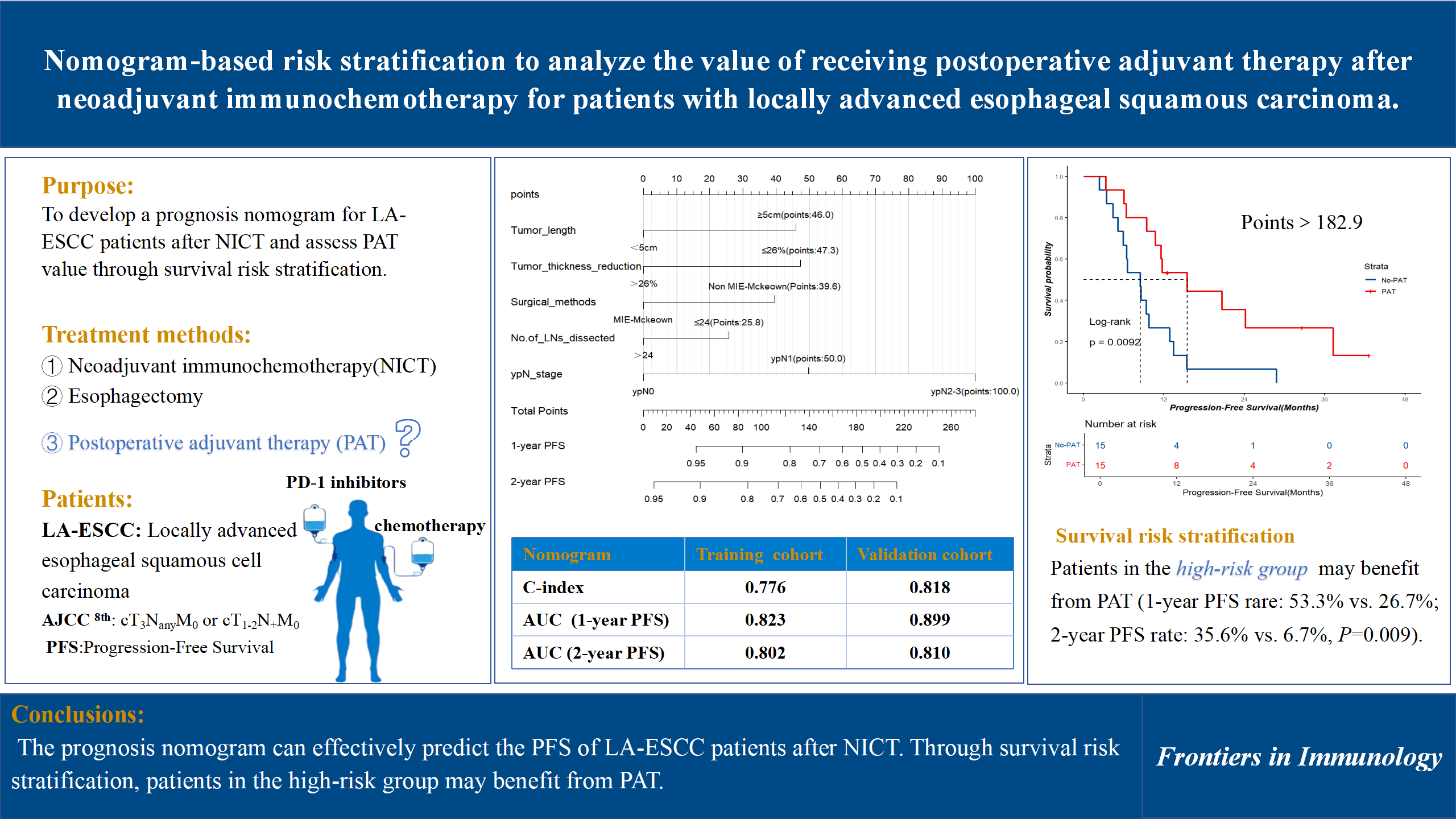

Supplement: Supplementary file 1 [file Image1.tif]
